# The Psychosocial Correlates of Non-suicidal Self-Injury Within a Sample of Adolescents With Mood Disorder

**DOI:** 10.3389/fpubh.2022.768400

**Published:** 2022-02-22

**Authors:** Linlin Meng, Diyang Qu, He Bu, Lijuan Huo, Ling Qi, Jiezhi Yang, Tiansheng Zheng, Xiangdong Du, Kongliang He, Yanni Wang, Yongjie Zhou

**Affiliations:** ^1^Linyi Mental Health Center, Linyi, China; ^2^Vanke School of Public Health, Tsinghua University, Beijing, China; ^3^Department of Social and Behavioural Sciences, City University of Hong Kong, Hong Kong, Hong Kong SAR, China; ^4^Affiliated Brain Hospital of Guangzhou Medical University, Guangzhou, China; ^5^School of Health Science and Nursing, Wuhan Polytechnic University, Wuhan, China; ^6^Shenzhen Health Development Research Center, Shenzhen, China; ^7^Kangning Hospital Affiliated to Wenzhou Medical University, Wenzhou, China; ^8^Suzhou Guangji Hospital, The Affiliated Guangji Hospital of Soochow University, Suzhou, China; ^9^Hefei Fourth People's Hospital, Hefei, China; ^10^Department of Maternal, Child and Adolescent Health, School of Public Health, Lanzhou University, Lanzhou, China; ^11^Shenzhen Mental Health Center, Shenzhen Kangning Hospital, Shenzhen, China

**Keywords:** psychosocial factors, non-suicidal self-injury, adolescent, mood disorder, self-esteem, family support

## Abstract

**Background:**

According to the integrated theoretical model, adolescents' behaviors were the outcome of the complex interplay between multiple levels. Non-suicidal self-injury (NSSI) is a serious and high prevalent problem among adolescents with mood disorders. However, a systematic perspective on psychosocial correlates among Chinese clinical adolescents is still rare.

**Method:**

The impact of several factors at the individual (i.e., sex, age, self-esteem, and psychological distress), family (i.e., family structure, family income, and family support), and social level (i.e., living environment, peer support, and teacher support) on the frequency of NSSI behaviors were investigated in the current study. This research included 621 Chinese adolescents with mood disorders from 20 hospitals.

**Results:**

Three-steps hierarchical regression analyses indicated that lower levels of psychological distress and higher levels of self-esteem were most associated with less frequency of NSSI behaviors. In addition, family support was negatively associated with the frequency of NSSI behaviors. After controlling the factors at individual and family levels, no significant association was found between the factors at the social level and the frequency of NSSI behaviors.

**Conclusion:**

These findings provide preliminary support for the notion that adolescent self-esteem and family support may effectively shield them from problematic behavior; nevertheless, adolescents suffering from more emotional pain can be even riskier. Thus, further intervention strategies should consider the non-independence of individual capacities, co-combinatory effects of mood disorder, and family environment in treating those vulnerable Chinese adolescents.

## Introduction

Non-suicidal self-injury (NSSI) has been defined as a direct self-harm behavior without death intention (1, 2) and is particularly dangerous during adolescence (3). NSSI is far more common in China than in Western countries (4), with 20–57% of community adolescents reporting at least one incident (5–7). Notably, NSSI is particularly frequent in adolescents with mood disorders, such as depression (8), anxiety disorder (9), bipolar disorder (10), and post-traumatic disorder (11), with a prevalence rate of up to 60% (12). In turn, this co-morbidity between mood disorders and NSSI may further increase the maladaptive psychological outcomes, such as suicidal behavior, which has attracted significant attention from researchers and practitioners (13). Thus, it becomes imperative to identify modifiable factors associated with NSSI, which are of great reference for developing preventive measures and plausible interventions for these vulnerable populations.

According to the integrated theoretical model (14) and socio-ecological framework (15), individuals' risk behavior is not a straightforward outcome of their own beliefs but a complex interplay between multiple levels, including individual, family, and social levels. The individual factors emphasize internal characteristics (e.g., capacities, mental health); whereas, family and social factors focus on social relationships or environments (e.g., family relationships, peer relationships, and teacher relationships). However, to the best of our knowledge, only a few studies have examined the potential factors of NSSI behaviors among Chinese clinical adolescents by using a systematic framework, which only offers a limited understanding of the NSSI behaviors.

### Individual-Level

At the individual level, mixed findings were observed for sex on the NSSI behaviors. For example, most studies showed that NSSI prevalence was 1.5–3 times higher in girls than boys (16, 17). This disparity may increase the clinical adolescent population (18), with girls significantly outnumbering boys (19). However, several studies suggest no significant relationship between sex and adolescents' self-harm behaviors (20, 21). Paradox findings were also found regarding the age difference. Barrocas et al. have recruited 655 American children aged from 7 to 16 years old. Their findings showed that older children report higher rates of NSSI than younger children (22). Nevertheless, these results were not replicated in a South African student sample (23). Taken together, there is still a need to clarify the sex and age difference in Chinese clinical adolescents' NSSI behavior, especially since the majority of the existing literature has been limited to community samples in the Western population (22).

There has been a link between the level of psychological distress and the NSSI behaviors (24–26). According to the emotional cascade model (27), NSSI would be served as a distraction strategy to rapidly reduce an individual's intensive negative feelings, leading to a sense of relief and even increasing their positive feelings. For example, Houben et al. found that individual with higher levels of negative emotion was more prone to NSSI behaviors (28). However, Hasking et al. indicated that psychological distress was not associated with NSSI frequency among students (29). Again, these inconsistent findings highlighted that more studies on the relationship between the level of psychological distress and NSSI frequency among our targeting population are required.

Moreover, some individual capacities negatively correlate with NSSI, including self-esteem (30). As explicitly noted by researchers, a lower level of self-esteem is often characterized by an overly harsh and negative attitude toward oneself (e.g., I am not a good or worthwhile person). In line with the experiential avoidance model (31), adolescents may engage in NSSI with a wish to escape from or avoid their unwanted poor psychological feelings (e.g., self-critical, negative attitudes). Self-esteem may also be an essential factor associated with Chinese clinical adolescents' NSSI frequency. Taken together, the first block of potential predictor factors in the present study involved individual factors, including sex, age, self-esteem, and psychological distress.

### Family Level

The supportive home environment (i.e., family support) may be a safeguard against engaging in NSSI for adolescents with emotional problems (32). Previous research has demonstrated that social support from family members can help community adolescents stop exhibiting NSSI (7, 33, 34). According to Bowlby (35, 36), inadequate parental support may exacerbate children's maladaptive coping techniques in the face of pressures, including suicide attempts (37). Thus, adolescents with emotional disorders may practice more NSSI behaviors to escape from the overwhelming and intolerable affect in the absence of supportive figures from their families.

Research on the familial structure has so far focused primarily on parent-child dyads, while remarkably little is known about siblings that adolescents were reported to spend more time with them than other family members (38). Siblings may act as protective factors against stressors or may further serve as triggers for more NSSI behaviors due to the interpersonal competition and conflicts (39), which is also an interesting thing to be confirmed in our study.

Several findings showed that lower family economic status was associated with more NSSI behaviors among adolescents. For example, a Swedish cohort study found that low parental socioeconomic status was associated with community adolescents' self-harm behaviors (40). However, a few research found no evidence for an association between family income and NSSI behavior, including Chinese adolescents (41) and Western adolescents with mood disorders (42). To clarify the findings on the frequency of NSSI behaviors within family contexts, the demographic characteristics (i.e., family income), family structure (i.e., only child or with siblings), and the level of family support were used in the second block as potential factors.

### Social Level

Prior research on the relationship between social environments and the frequency of NSSI behaviors in adolescents, especially those with a mood disorder, has been limited. Adolescents' adaptive outcomes are linked to their relationships with peers and teachers (43). For example, a previous study found that the quality of peer connections is strongly associated with children's internalizing difficulties (44), which are, in turn, positively associated with NSSI behaviors (13). Furthermore, adolescents who feel supported by their teachers are less likely to engage in NSSI behavior (45–47).

Chinese economic and cultural systems have differentiated rural and urban areas. Generally, the economic development level is relatively lower in rural regions, and cultural norms are more conservative (17), limiting their schema on NSSI. It's also possible that residing in a remote area with fewer social resources (e.g., social services, mental health clinics, and school counseling centers) would be a risk factor for the frequency of NSSI behavior. Plener et al. (48), on the other hand, discovered no difference in NSSI frequency between people living in German cities and those living in rural regions. Therefore, the third block contained three possible predictors: living environment, peer support, and teacher support.

### The Present Study

It is still unclear to what extent the prior findings among western adolescents can be generalized to clinical adolescents in China and whether any specific factor contributes to more frequent NSSI behaviors in this population. Thus, the purpose of this study was to investigate the association between those psychosocial factors above from individual level, family level to a social level, and the frequency of NSSI behaviors among adolescents with mood disorders.

## Methods

### Participants

A three-stage sampling procedure was adopted in this investigation. To begin, nine provinces were chosen to recruit cooperative hospitals based on the economic situation of each province in China as measured by the good, medium, and general economy levels. These samples represent the diversity of geography, economic development, and public health resources. Among 20 hospitals from nine provinces, convenience sampling was used to enroll adolescents with mood disorders.

The data was collected from September 2020 to November 2020. Participants had to meet the following criteria to be included: (a) had engaged in at least one non-suicidal self-injury behavior in the past year; (b) have been diagnosed with a mental disorder by senior psychiatrists using the Diagnostic criteria and Statistical Manual of Mental Disorders Fourth Edition (DSM-IV-TR); (c) aged 12–18 years old. In addition, adolescents who were unable to complete the survey (for example, severe physical illness and cognitive impairment) were excluded from this study. The research's introduction and consent forms were distributed to participants and their legal guardians prior to the start of the study. Written informed consent was obtained from the participants and their legal guardians. The participants did not receive any incentive. Before the study, ethical approval was granted. The final sample included 621 adolescents: 93 boys and 528 girls. On average, participants were 15 years old (SD = 1.7, range = 12–18). In total, 32% of participants lived in cities, while 68% lived in rural areas. The vast majority (70%) of participants had siblings.

### Measures

Demographic data were collected, including sex (i.e., 0 = boys; 1 = girls), age, family structure (i.e., 0 = have siblings, 1 = do not have siblings), family income (i.e., 0–80,000 CNY yearly coded as 1; 80,000–200,000 CNY yearly coded as 2; 200,000–300,000 CNY yearly coded as 3; over 300,000 CNY yearly coded as 4) and living environment (i.e., 1 = living in rural areas, 2 = living in cities).

#### NSSI

The Functional Assessment of Self-Mutilation (FASM; Lloyds, 1997) is a self-reported questionnaire that assesses the techniques, frequency, and function of self-mutilation. Participants were asked if they participated in any of the 11 different types of NSSI and how often they did so.

Experts advised that NSSI behavior (such as punching walls or objects) be included because it is highly frequent among adolescents (49). Following the procedure used in the previous study (1, 50), the frequency scores were recoded into a 5-point scale, indicating the frequency of the NSSI in the previous 12 months: 1 (0 times), 2 (1 time), 3 (2–5 times), 4 (6–10 times), and 5 (≥11 times). Total scores of these 12 items were used, with higher scores indicating a higher level of NSSI frequencies. Cronbach's alpha was 0.807 in this study.

#### Self-Esteem

The 10-item Rosenberg Self-Esteem Scale (RSES; Rosenberg, 1965) was used. The scale has been widely used among Chinese children and adolescents (8, 51). A high score denotes a strong level of self-esteem. The Cronbach's alpha in this study was 0.849, showing good internal consistency.

#### Family Support, Peer Support, and Teacher Support

The 12-item Multidimensional Scale of Perceived Social Support [MSPSS; (52)] was used to assess three sources of support: *Family, Friends*, and *Significant others*. In this study, the term *Significant others* were replaced by teachers to adapt to the target population. A higher score indicates a higher level of perceived social support. The Chinese version of this scale has been used in Chinese adolescents, and all subscales demonstrate good internal consistency (53). In the current study, the Cronbach's alphas of the three subscales were above 0.83, showing good internal consistency.

#### Psychological Distress

Kessler Psychological Distress Scale was used [K-10; (54)]. A higher score indicates a higher level of psychological distress. The Chinese version has been used in children and demonstrates good psychometric properties (55). In the current study, Cronbach's alpha was 0.89, showing good internal consistency.

### Statistical Analysis

All statistical analyses were performed using IBM SPSS 28.0. Categorical variables were dummy-coded. First, the means and standard deviations were compared by *t*-tests to explore the difference of gender, family structures, and living environments in the frequency of NSSI behaviors. Next, the bivariate and point biserial correlations were examined to test the associations among variables.

Then, the hierarchical regression analyses were performed to investigate the effects of different psychosocial factors on the frequency of NSSI behaviors. The potential factors involved in the individual level, including sex, age, self-esteem, and psychological distress, were entered in the first block. After controlling for variables in the first block (level 1), the family structure (i.e., only child or with siblings), family income, and family support were entered in the second block (level 2). The living environment (i.e., rural area or cities), peer support, and teacher support were input in the third block while controlling for variables in the first and second blocks (level 3). Given the multiple variables, adjusted *p*-values using the Benjamini and Hochberg (56) false discovery rate correction were calculated to maintain the Type I error rate below 0.05 and are reported in **Table 2**.

## Results

“Cut or carved on skin” was the most common NSSI behavior occurrence (90%, M = 2.72, SD = 1.2); “Burned your skin” was the least common (9%; M = 0.19, SD = 0.65). Girls (85%, M = 15.89, SD = 9.18) reported a considerably greater frequency of NSSI behavior than boys (15%, M = 13.11, SD = 9.53; *t* = 2.67, *p* = 0.008). In addition, the only child in the family (30%, M = 14.21, SD = 9.09) showed a less rate of NSSI behaviors than the child with siblings (70%, M = 16.00, SD = 9.32; *t* = 2.21, *p* = 0.03). However, there was no difference in the frequency of NSSI behavior among adolescents from cities (32%, M = 16.07, SD = 9.53) or rural areas (68%, M = 15.19, SD = 9.16; *t* = 1.10, *p* = 0.21).

Table 1 shows descriptive statistics and bivariate correlations among the variables. Sex, age, self-esteem, psychological distress, family structure, family support, peer support, and teacher support were correlated with the frequency of NSSI behaviors (*p* < 0.05).

**Table 1:** Descriptive statistics and correlations among study variables (*N* = 621).

	***M (SD)***	**1**	**2**	**3**	**4**	**5**	**6**	**7**	**8**	**9**	**10**
1. Sex	-	-									
2. Age	15 (1.65)	−0.12^**^	-								
3. Self-esteem	22.86 (6.92)	−0.07	0.17^***^	-							
4. Psychological distress	36.32 (7.46)	0.12^**^	−0.07	−0.55^***^	-						
5. Family structure	-	0.02	0.07	0.09^*^	−0.06	-					
6. Family income	-	−0.11^**^	0.01	0.07	−0.03	0.07	-				
7. Family support	14.84 (5.60)	−0.01	0.23^***^	0.32^***^	−0.21^**^	0.07	0.0004	-			
8. Living environment	-	0.06	0.08^*^	−0.08	0.04	−0.16^***^	−0.25^***^	−0.05	-		
9. Peer support	15.93 (6.19)	−0.02	0.13^**^	0.26^***^	−0.16^**^	−0.04	0.09^*^	0.22^***^	0.01	-	
10. Teacher support	12.21 (5.70)	−0.05	0.02	0.25^***^	−0.17^**^	0.01	0.01	0.26^***^	−0.04	0.27^***^	-
11.NSSI behavior	-	0.11^**^	−0.09^*^	−0.40^***^	0.44^***^	−0.09^*^	−0.02	−0.25^***^	0.04	−0.14^***^	−0.16^***^

* *p < 0.05*.

** *p < 0.01*.

*** *p < 0.001*.

The findings of the hierarchical regression analysis are shown in Table 2. The results revealed that only block 1 and block 2 contributed significantly to the regression model, and these variables at the individual and family level explained 24% of the total NSSI behaviors variance. A higher level of self-esteem remained significantly associated with a lower frequency of NSSI (*B* = −0.25, *SE* = 0.06, β = −0.19, *p* < 0.001). Psychological distress also showed a substantial positive effect on the frequency of NSSI (*B* = 0.38, *SE* = 0.05, β = 0.30, *p* < 0.001). Other demographic characteristics (i.e., sex and age) had no bearing on the occurrence of NSSI. Perceived social support from family had a negative impact on the frequency of NSSI at the family level (*B* = −0.20, *SE* = 0.06, β = −0.01, *p* = 0.01). None of the other factors (i.e., siblings, income) were significantly associated with the frequency of NSSI. At the social level, factors (i.e., rural/local cities, perceived support from peers, perceived support from teachers) did not significantly correlate with the frequency of NSSI.

**Table 2:** Hierarchical regression analyses of associator factors of NSSI behaviors (*N* = 621).

	**Model 1**					**Model 2**					**Model 3**					
	**B**	**SE**	**β**	***T***	***p***	**B**	**SE**	**β**	***t***	***p***	**B**	**SE**	**β**	***t***	***p***	**Adjusted *p***
**Individual level**																
(Constant)	8.98	4.08		2.20	0.03	9.18	4.11		2.23	0.03	9.77	4.27		2.29	0.02	0.06
Sex	1.28	0.93	0.05	1.37	0.17	1.46	0.93	0.06	1.56	0.12	1.43	0.94	0.06	1.53	0.13	0.29
Age	−0.13	0.20	−0.02	−0.63	0.53	0.02	0.21	0.00	0.09	0.93	0.01	0.21	0.00	0.06	0.95	0.95
Self-esteem	−0.30	0.06	−0.22	−5.15	<0.001	−0.25	0.06	−0.19	−4.25	<0.001	−0.24	0.06	−0.18	−3.94	<0.001	<0.001
Psychological distress	0.39	0.05	0.31	7.34	<0.001	0.38	0.05	0.30	7.16	<0.001	0.37	0.05	0.30	7.12	<0.001	<0.001
**Family level**																
Family structure						−0.94	0.72	−0.05	−1.31	0.19	−0.98	0.73	−0.05	−1.34	0.18	0.33
Family income						0.08	0.38	0.01	0.20	0.84	0.10	0.39	0.01	0.26	0.80	0.95
Family support						−0.20	0.06	−0.01	−3.26	0.01	−0.19	0.06	−0.11	−2.94	0.003	0.01
**Social level**																
Living environment											0.05	0.74	0.00	0.07	0.95	0.95
Peer support											−0.03	0.06	−0.02	−0.53	0.59	0.81
Teacher support											−0.05	0.06	−0.03	−0.77	0.44	0.69
*R* ^2^	0.23					0.25					0.25					
Adjusted *R*^2^	0.23					0.24					0.24					
*F* for *R*^2^ change	46.23					4.21					0.36					
*p*-value	<0.001					0.01					0.78					

*Sex (0 = boys; 1 = girls), Age, family structure (0 = have siblings, 1 = do not have siblings); Family support, perceived social support from family; Environment, living environment (1 = living in rural areas, 2 = living in cities); Peer support, perceived social support from peer; Teacher support, perceived social support from teacher*.

## Discussion

The present study examined the effects of different psychosocial factors across multiple levels on the frequency of Chinese clinical adolescents' NSSI behaviors. Higher self-esteem and lower psychological distress can reduce the risk of adolescents' engaging in the higher frequency of NSSI behaviors. Adolescents who reported receiving more family support were less likely to engage in NSSI behaviors.

### Individual-Level

Self-esteem was the strongest protective factor for the frequency of NSSI behaviors among adolescents with mood disorders. Specifically, adolescents with higher self-esteem were less likely to participate in NSSI behaviors. The findings were consistent with the prior research among Western inpatient adolescents (57) and a non-clinical sample of Chinese adolescents (8, 58). There are two possible explanations. According to the Experiential Avoidance Model (31), adolescents with poor self-esteem have more hostile, critical, and unpleasant self-perception types, making them more likely to engage in problematic behaviors (e.g., NSSI) to avoid or cope with distressing inner states (57). Second, people with poor self-esteem struggle with body awareness, which can lead to a lack of feelings when it comes to inflicting physical pain on themselves, leading to increased self-injury behaviors (59, 60). More research is needed to clarify the underlying mechanism between self-esteem and NSSI behaviors.

In accordance with earlier research among Western adolescents, psychological distress was the strongest risk factor for the NSSI frequency among adolescents (61). The most popular models of NSSI conceptualized it as an emotional regulation strategy (27, 62). For example, (63) reported that inpatient adolescents used NSSI behaviors to reduce their dysphoric affect. Thus, it is likely that Chinese adolescents with mood disorders who experience more psychological distress than community samples may also engage NSSI to cope with their excessive emotional distress (62). Therefore, intervention programs to teach adaptive emotional regulation strategies for clinical adolescents who experience higher levels of psychological distress may also reduce their engagement in NSSI (64).

### Family Level

For the family-level factors, consistent with previous research, we found that family support was a protective factor for adolescents' engaging in NSSI behavior and vice versa (58). A possible explanation is that adolescents with poor familial support systems perceive their parents as untrustworthy, feeling unworthy of being cared for or loved (65). Specifically, by operating fundamental psychological defense mechanisms unconsciously, adolescents may internalize and experience the hostile image of their parents as self-hatred to reduce their painful feelings (65, 66), in turn, be more inclined to engage NSSI as a self-punishment (61).

Alternatively, NSSI was more likely to serve as a strategy to escape from the intolerable effect without more adaptive mechanisms. Given the attachment theory (36, 67), a high-quality family support system may foster the development of children's emotion regulation skills. For instance, parents provide the fundamental exemplary model, and their children learn how to express rather than repress their negative feelings and regulate emotions by adaptive strategies from their parents (68, 69), for example, crying or asking for help (70).

Another possible explanation is that those adolescents may engage in NSSI to “get a reaction from parents” (1, 62). In a large sample of Chinese high school students, You et al. subsequently found that “social influence,” including “get parents to understand or notice me” and “to get others' attention,” were rated as secondary reasons for NSSI among (71). A helpful and friendly family appears to be a key defense against NSSI; according to all prior research, the result was consistent with previous studies of western adolescents (34).

### Social Level

The adolescent is considered to be easily influenced by the social environment, including peer and teacher, and living environment. Interestingly, we found no significant association between social environments and NSSI among those Chinese adolescents with mood disorders. This finding is inconsistent with previous Western findings (72, 73). A possible explanation was that the poor living environment, including relationships with family, peers, and teachers might influence those adolescents' problematic behaviors *via* individual capacities, such as self-esteem. In line with the Multi-System Model of Resilience (74), the external resources would contribute to individuals' internal resources, thereby promoting their adaptive outcomes. For example, Thompson et al. found that self-esteem was a significant mediator between peer relationships and adolescents' internalizing, externalizing, and delinquent behaviors (75). Additionally, it has also been found that adolescents who feel supported by their teacher may increase their sense of self-worth, predicting more emotional adjustment and less psychological maladjustment (76).

To further test this hypothesis, we used the mediation model. Interestingly, self-esteem was revealed to have a significant mediating effect between family, peer, teacher support, and NSSI behavior frequency. Further research should employ a longitudinal design to understand better how social environments affect the frequency of those Chinese adolescents' NSSI behavior.

### Limitations and Implications

The study has several limitations. First, it should be acknowledged that all the measures in this study are self-reported, which may be an artifact of shared method variance. Future research using physiological measures, such as stress hormones, pain tolerance, may provide a more comprehensive perspective on NSSI. Second, the intersection of NSSI and sex has been limited by using small samples of boys drawn primarily from clinical populations. Future research designs should be more deliberate in selecting samples. Third, correlational and cross-sectional would limit us from drawing any causal conclusion, further producing biased estimates. Thus, a prospective, longitudinal design is required to tases the temporal order between variables overtimes. Fourth, clinical samples were obtained from different China regions, which helps represent the nation. However, there exist different cultural norms, social-economic levels, and other social characteristics. Thus, a region-level residual in multilevel analyses should be further considered (77). Fifth, individuals with suicidal ideation may employ different functions than individuals without suicidal ideation, which should be further explored. Finally, the underlying mechanism from social support to NSSI behaviors *via* self-esteem could be further examined in future studies. Despite these limitations, this study has examined the related psychosocial determinates (individual, family, and environmental factors) among a clinical sample of Chinese adolescents, which provides a starting point for further intervention studies.

Our findings can help health professionals design the prevention and intervention programs among adolescents engaging in NSSI with the comorbid mental disorder under the Chinese context. Integrated interventions targeting low self-esteem and high psychological distress, such as the compassion-focused treatment (including self-compassion and emotional regulation), may help achieve more with less effort (78). Treating those adolescents with respect and dignity was also necessary. Second, the NSSI prevention and intervention program should provide parents with better tools to help their children. Specifically, helping educated parents improve communication, parenting skills, and knowledge of NSSI might be a promising direction for minimizing the possibility of adolescents engaging in NSSI. Thirds, the school-based intervention programs may significantly improve children's poor maladaptive strategies *via* strengthening their self-esteem (79).

## Data Availability Statement

The raw data supporting the conclusions of this article will be made available by the authors, without undue reservation.

## Ethics Statement

The studies involving human participants were reviewed and approved by the Human Research Ethics Committee of Shenzhen Kanning Hospital [2020-K021-01]. Written informed consent to participate in this study was provided by the participants' legal guardian/next of kin.

## Author Contributions

YZ developed the study concept. LM collected the data, oversaw the data collection, and organized the database. DQ developed the study design, performed the data analysis and interpretation, and drafted the manuscripts with support from all authors. HB performed the data analysis and interpretation. LM, HB, LH, YW, and YZ provided critical revisions and provided logical suggestions. LQ contributed to the data analysis and study design. JY organized the database. JY, TZ, XD, and KH collected the data. All authors contributed to the article, read, and approved the submitted version.

## Funding

The study was sponsored by Sanming Project of Medicine in Shenzhen No. SZSM202011014. Shenzhen Fund for Guangdong Provincial High-level Clinical Key Specialties No. SZGSP013. Shenzhen Key Medical Discipline Construction Fund No. SZXK072 and Shenzhen Science and technology research and Development Fund for Sustainable development project No. KCXFZ20201221173613036.

## Conflict of Interest

The authors declare that the research was conducted in the absence of any commercial or financial relationships that could be construed as a potential conflict of interest.

## Publisher's Note

All claims expressed in this article are solely those of the authors and do not necessarily represent those of their affiliated organizations, or those of the publisher, the editors and the reviewers. Any product that may be evaluated in this article, or claim that may be made by its manufacturer, is not guaranteed or endorsed by the publisher.
